# Association of lean body mass to menopausal symptoms: The Study of Women's Health Across the Nation

**DOI:** 10.1186/s40695-020-00058-9

**Published:** 2020-09-15

**Authors:** Rosanne Woods, Rebecca Hess, Carol Biddington, Marc Federico

**Affiliations:** Department of Exercise Science and Sport Studies, California University of Pennsylvania, 50 University Ave, California, PA 15419 USA

**Keywords:** Lean body mass, Vasomotor symptoms, Hot flashes, Menopause - women

## Abstract

**Background:**

The purpose of this study was to examine the association of lean body mass (LBM) to the development of vasomotor symptoms (VMS) as women transition through menopause.

**Methods:**

This study is a secondary use of data available for public use from follow up visits six through 10 for participants in the Study of Women’s Health Across the Nation. The study examined 2533 women, between the ages 42–52 years, each year over a10-year period. Data was modeled for associations of lean body mass and VMS. Changes in LBM since prior visit and since baseline were also modeled along with differences in means using binary logistic regression, adjusting for covariates.

**Results:**

LBM was significantly associated to concurrent VMS (*p* = .036), percent change in LBM since prior visit (*p* = .003), percent change since baseline (*p* < .001), and overall means associations (*p* = .023). LBM was not significant for VMS at individual visit measures. In mixed regression modeling, time was significant (*p* < .0001) at all visits. The estimated probability of developing VMS decreases significantly as LBM increases.

**Conclusions:**

Lean body mass is negatively associated with incident VMS. Our data suggests that maintaining higher levels of LBM during the menopausal transition may be protective against the development of VMS. Every woman will experience menopause in her life and the ability to potentially prevent the onset of specific symptoms through basic interventions, such as resistance training to increase lean body mass, may positively impact this large population.

## Introduction

Menopause is a significant event in many women’s lives as it marks the end of the natural reproductive life. For most women, menopause will occur between the ages of 40 and 58 years with the average being 51 years [1]. About 80–96% of women experience mild to severe physical or physiological menopause-related complaints as they approach menopause due to declining estrogen levels [2]. Symptoms may include hot flashes and night sweats, depression, irritability, sleep disorders, increased abdominal fat mass, increased prevalence of metabolic syndrome, and increased risk of cardiovascular disease [3]. Hot flashes and night sweats are the most common symptoms of menopause and are collectively referred to as vasomotor symptoms (VMS). It is reported that 60–80% of women will experience VMS at some point during the menopausal transition [4].

As they transition through menopause, women will simultaneously experience a decrease in basal metabolic rate and a loss of lean muscle tissue which increases the risk of weight gain and obesity [5]. Sarcopenia develops and is highly prevalent during menopause and is primarily due to an imbalance between muscle protein synthesis and breakdown, contributed to by an increase in oxidative stress, pro-inflammation markers, and hormonal changes [6]. Evidence indicates that muscle strength and quality (ratio of muscle strength to mass) may be negatively associated with the severity of menopausal symptoms due to declining levels of sex hormones and the resulting increase in oxidative stress [7]. Postmenopausal women have been shown to have significantly higher oxidative stress blood marker levels and lower antioxidant capacity relative to premenopausal women [8].

For postmenopausal women, chronic systemic inflammation, oxidative stress, abdominal visceral adipose tissue, dyslipidemia, sarcopenia, and a sedentary lifestyle are all risk factors for metabolic syndrome [9]. A systematic review found that the menopausal transition is associated with a decline in estrogen, growth hormone, insulin-like growth factor (IGF-1), and dehydroepiandrosterone (DHEA), a decrease in muscle protein synthesis, and an increase in catabolic factors such as the pro-inflammatory cytokines, and tumor necrosis factor alpha (TNF-**α**) or interleukine 6 (IL-6) [10]. A recent study found that weight-adjusted lean body mass (LBM) and skeletal muscle area were protective against weight-associated insulin resistance and metabolic abnormalities [11] suggesting that women with lower muscle mass and fewer estrogen receptors are therefore at greater risk for metabolic complications [12]. Decreased LBM has been found to be the most important contributor to changes in metabolism for postmenopausal women as it correlates to low whole-body fat oxidation and energy expenditure which in turn are associated with high visceral fat mass and low insulin resistance [13]. Maintaining adequate levels of muscle mass as women transition into menopause may play a role in minimizing the risks of sarcopenic obesity and protect against the development of deleterious metabolic conditions commonly associated with menopause. However, little is known regarding the role of LBM and its influence on menopausal symptoms throughout the transition period. The following hypotheses were examined: Hypothesis 1 (H_1_) - Lower concurrent LBM will be associated with greater concurrent incident reporting of VMS; Hypothesis 2 (H_2_) - In longitudinal analyses, lower LBM over time, since baseline, will be associated with greater incident reporting of VMS; Hypothesis 3 (H_3_) - In longitudinal analyses, lower LBM over time, since last annual visit, will be associated with greater incident reporting of VMS.

## Methods

This study was a secondary analysis of existing data utilizing the public dataset from the SWAN study [14–19]. Details of the SWAN design and recruitment procedures are reported elsewhere [20], however, a brief summary is provided here. Baseline eligibility criteria included being aged 42 to 52 years, having a uterus and at least one ovary, not being pregnant or lactating, not using oral contraceptives or hormone therapy in the previous 3 months, and having at least one menstrual cycle in the preceding months. Participants self-identified as African-American (28%), Caucasian (47%), Chinese (8%), Hispanic (8%), or Japanese (9%). Eligible women meeting the inclusion criteria were invited to join the cohort and were seen within 3 months of the initial survey for their baseline assessment where a written informed consent was obtained. Assessments consisted of questionnaires regarding medical history, medication, menstrual history, lifestyle, psycho-social factors, physical and psychological symptoms, and health-related quality of life, as well as blood and urine specimen collection and physical measures. Procedures specific to this study included annual examinations, questionnaires, and bioelectrical impedance analysis measures. Approval for this study was granted by the Institutional Review Board at California University of Pennsylvania.

### Anthropometry/body composition

Analysis was limited to the use of body composition data collected using BIA and included 2533 women. BIA is based on measurement of the transmission speed of a one-quarter volt electrical pulse between electrodes attached at the feet and electrodes attached across the knuckles of the hand. Because fat-free mass is comprised of water, proteins, and electrolytes, conductivity is greater in fat-free mass than in fat mass [21]. Resistance and reactance are used to estimate total body water, and by extension, fat mass and lean mass, with the latter including bone [22]. The validity and predictive value of BIA in menopausal women has been confirmed by a recent study [23]. Skeletal muscle mass was calculated by the method of Janssen et al. [24], who subsequently indexed skeletal muscle mass to height for a skeletal muscle index (SMI = skeletal muscle mass (kg) / height (m^2^)). For the purposes of this study, LBM refers to the level of skeletal muscle mass and will be represented by the variable SMI. Fat free mass, total body water, and percent body fat were all provided by RJL Systems and validated using NHANES III data [25]. Fat free mass (kg) and fat mass (kg) were both indexed to height to create fat mass index (FMI = kg/m^2^) and fat free mass index (FFMI = kg/m^2^). Height (m) and weight (kg) were measured in light clothing, without shoes, using a standard protocol with a stadiometer for height and a balance beam scale for weight. Hip circumference (cm) was measured at the iliac crest and waist circumference (cm) was measured at the level of the natural waist or the narrowest part of the torso from the anterior aspect [26, 27].

### Vasomotor symptoms

Hot flashes and night sweats were assessed via questionnaire at each SWAN visit. Women responded to two questions that separately asked them to record how often hot flashes and night sweats were experienced in the 2 weeks prior to the annual visit (not at all, 1–5 days, 6–8 days, 9–13 days, everyday). Accuracy of recall for VMS among the SWAN participants was previously verified [28].

### Covariates

Covariates were selected on the basis of previously documented associations with VMS [29] and body composition [27], and included age, educational level (less than high school, high school, some college, college, or post baccalaureate degree), race/ethnicity, quality of life, and menopausal transition stage. Race/ethnicity and educational level were self-reported in the SWAN screening interview. SWAN participants were assessed for menopausal status assignment based on annual reports about menstrual bleeding and its regularity. Pre-menopause was identified as no decreased regularity in menstrual bleeding during the last year. Other classifications were early perimenopause (decreased menses in previous 3 months), late perimenopause (no menses for 3–11 months), and postmenopause (no menses for 12+ months) [30]. Surgical menopause was defined by report of either hysterectomy or oophorectomy, and hormone therapy (HT) use was reported as use of HT during the year [30]. The Medical Outcomes Short-Form 36 (SF-36) was used to assess health related quality of life (HRQL) using the original coding algorithm in which raw scores are transformed to a 0 to 100 range.

### Statistical analysis

Characteristics of the sample were described by means (standard deviation) and frequency (%). At baseline, two VMS groups – any or none – were compared for group differences in, and associations among, demographics (age, race/ethnicity, education), quality of life (SF-36 score), and clinical characteristics (weight, hip and waist circumference, menopausal status, fat mass, fat free mass, skeletal mass), and VMS was estimated using chi square test (*x*^2^) for categorical variables, and Kruskal-Wallis test for continuous variables. A scatter plot matrix was used to examine linear correlations among variables. For the purposes of modelling, LBM is represented by the SMI variable. Additionally, to account for the nonindependence of longitudinal observations derived from the same woman and data in which the number of observations may differ across women, longitudinal modeling using SAS PROC MIXED incorporated a random intercept term to account for the correlated errors among repeated measures of the same woman. Missing values of time-varying variables were interpolated based on prior and subsequent values for gaps of one to two visits as in previous SWAN analyses [31]. To assess H_1,_ incident VMS was modeled as a function of concurrent LBM using logistic regression analysis. To address H_2_ regarding long term change in LBM, the model was expanded to add within-woman percent change in LBM since baseline and to address H_3,_ regarding recent change in LBM, the model was expanded to add within-woman percent change in LBM since prior visit (approximately 1 year earlier). The overall association between LBM and VMS was estimated in binary logistic regression models. Statistical analyses were one-tailed with an alpha level of 0.05 and conducted using SAS University Edition (© 2012–2018, SAS Institute Inc., Cary, NC).

## Results

At baseline (visit 6) there were 2533 participants remaining in the SWAN study who were on average 52 years old and the differences in the group characteristics are shown in Table 1. Only women reporting no symptoms at baseline (*n* = 1179) were included in longitudinal analyses for VMS with LBM. At visit 10 there were 800 women remaining in the study.

**Table 1:** Baseline Characteristics by symptom status, SWAN

	Presence of vasomotor symptoms (VMS)				
	*None*	(% *n*)	Any	(% n)	*p*
***n***	1179	(47%)	1354	(53%)	
**Age, yr (mean ±)**	52.0 ± 2.7		52.0 ± 2.6		
**Race/Ethnicity (*n*)**					< .0001
African American	272	(36%)	494	(64%)	
Caucasian	619	(50%)	622	(50%)	
Chinese	118	(52%	107	(48%)	
Hispanic	144	(54%)	122	(46%)	
Japanese	26	(67%0	13	(33%)	
**Education (*n*)**					< .0007
≤ High School	225	(44%)	286	(56%)	
Some College	349	(42%)	481	(58%)	
≤ College Graduate	595	(50%)	584	(50%)	
**Menopausal Status (*n*)**					< .0001
Postmenopausal	430	(42%)	591	(58%)	
Late Perimenopausal	*77*	(32%)	163	(68%)	
Early Perimenopausal	368	(50%)	372	(50%)	
Premenopausal	54	(68%)	26	(33%)	
**Body Composition (mean ± SD**)					
Weight (kg)	74.3 ± 20.7		78.4 ± 20.7		<.0001
BMI (kg/m^2^)	28.1 ± 7.3		29.6 ± 7.3		<.0001
Body Fat (%)	36.8 ± 8.0		39.1 ± 7.6		<.0001
Total Body Fat (kg)	28.3 ± 13.2		31.9 ± 13.9		<.0001
Fat Mass Index (kg/m^2^)	10.75 ± 4.9		12.03 ± 5.11		<.0001
Fat Free Mass (kg)	45.1 ± 7.4		46.3 ± 7.6		.0002
Fat Free Mass Index (kg/m^2^)	17.1 ± 2.4		17.5 ± 2.5		.0003
Skeletal Muscle Mass (kg)	20.6 ± 3.3		20.9 ± 3.3		.0423
Skeletal Muscle Index (kg/m^2^)	7.8 ± 1.1		7.9 ± 1.1		.1298
Waist Hip Ratio (%)	0.81 ± 0.07		0.83 ± 0.07		<.0001

Notes: *SWAN* Study of Women’s Health Across the Nation, *BMI* Body mass index;

At baseline, SMI showed a strong positive correlation to FFMI both for symptoms = none (r_0_ (864) = 0.931, *p* < .0001) and symptoms = any (r_1_ (1143) = 0.933, *p* < .0001), and a moderate positive correlation to FMI (r_0_ (864) = 0.567, *p* < .0001) (r_1_ (1143) = 0.579, *p* < .0001). FMI showed a strong positive correlation to FFMI at baseline for both groups (r_0_ (864) = 0.820, *p* < .0001; r_1_ (1143) = 0.826, *p* < .0001). Pearson correlation of mean SMI to mean FMI (calculated on a participant basis over visits 6–10) was moderately strong for ‘none’ (r_0_ (405) = 0.648, *p* < .0001) and relatively weak for ‘any’ with (r_1_ (611) = 0.559, *p* < .0001). Mean FFMI was strongly correlated to mean FMI in both groups (r_0_ (405) = 0.860, *p* < .0001; r_1_ (611) = 0.829, *p* < .0001). Mean SMI was very strongly correlated to both symptom groups for mean FFMI (r_0_ (405) = 0.942, *p* < .0001; r_1_ (611) = 0.923, *p* < .0001). SMI continued to be very strong as expected and the relationship of FFMI to FMI remained consistent over time.

Models were developed to address the three hypotheses and are presented as odds ratios and 95% confidence intervals for incident VMS in Table 2. Adjusted models included time-varying covariates of FMI, age, and menopausal status and single-time variates of race/ethnicity and education. Quality of life scores were not significant in any models and were not considered in further analyses. For H_1_ (VMS associations with concurrent LBM) results are summarized in Model 1 where LBM is significant in both the unadjusted (*p* < .0001) and adjusted (*p* = .036) analyses. For H_2_ (VMS associations with LBM over time since baseline), within woman percentage change since baseline was added to Model 1 and results are presented as Model 2. Again, LBM showed significance in both unadjusted (*p* = .001) and adjusted (*p* < .001) modelling. For H_3,_ (VMS associations with LBM over time since prior visit) within women percentage change since prior visit was added to Model 1 and the results are summarized as Model 3. LBM remained significant in unadjusted (*p* = .009) and adjusted (*p* = .003) models respectively.

**Table 2:** Association of VMS to lean body mass (Odds Ratios)

	Unadjusted			Adjusted *		
SMI/FFMI	*OR*	95% CI	*p*	OR	95% CI	*p*
Model 1^a^	0.54	[0.43, 0.68]	<.0001	0.93	[0.87, 0.99]	.036
Model 2^b^	0.15	[0.05, 0.41]	.001	0.12	[0.04, 0.33]	<.001
Model 3^c^	0.21	[0.08, 0.58]	.009	0.17	[0.06, 0.49]	.003
Model 4^d^	0.38	[0.18, 0.76]	.007	0.22	[0.08, 0.61]	.023

Note: *VMS* Vasomotor symptoms, *OR* Odds ratio, *CI* Confidence interval, *LBM* Lean body mass, *SMI* Skeletal muscle index, *FFMI* fat free mass index;

^a^VMS with concurrent LBM

^b^Model 1 plus % change in SMI/FFMI since baseline

^c^Model 1 plus % change in SMI/FFMI since prior visit

^d^Overall association of mean SMI, mean FFMI, mean FMI, mean VMS

^e^Adjusted for covariates fat mass index, age, race/ethnicity, education, menopausal status

**Fig. 1:**
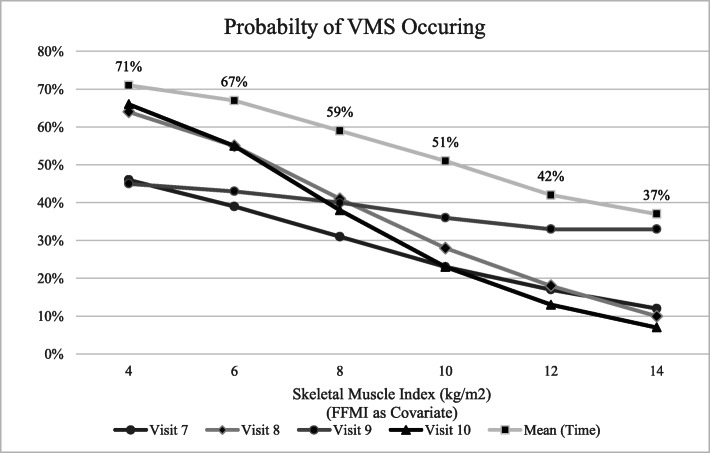
Predicted probability of symptoms occurring at given levels of SMI (adjusted for FFMI and FMI) in women with no VMS at visit 6 (baseline)

FMI was significant in adjusted models 1 (*p* = .0003), 2 (*p* < .0001), 3 (*p* < .0001), and 4 (*p* = .0073). Race/ethnicity was significant in Models 1 (*p* < .0001) and 2 (*p* = .0139) but failed to reach significance for Model 3 or 4. Menopausal status was significant (*p* = .0052) in Model 4 only. Age was not significant in any model. In mixed regression models of VMS and LBM repeated for time (symptoms as random effect), results were significant (*p* < .0001) for least squares means at visit (time) 6, 7, 8, 9, and 10. Additionally, significance (*p* < .0001) was found for the difference of least square means (Tukey-Kramer method) at visit 6 (time effect 6–7, 6–8, 6–9, 6–10), and for time effect visit 8–10 (*p* = .007) (data not shown). Further, estimated predicted probabilities were examined to determine the likelihood of LBM to predict the development of VMS. The probabilities estimated for given levels of SMI at each of the four visits following baseline, including the mean over time, for women reporting no VMS at visit 6 (*n* = 1179) shows significant negative correlation to levels of SMI (Fig. 1).

## Discussion

This study is among the first to examine the relationship of lean body mass to vasomotor symptoms longitudinally. Using the data for this large, multiethnic sample of mid-age women from the SWAN study, we found that participants with higher relative levels of LBM were less likely to develop VMS as they transitioned through menopause. This effect was found to be independent of sociodemographic factors and levels of fat mass. Additionally, as the average age of the participants in our study increased from 52.0 to 56.6 years and the number of women who were postmenopausal increased from 40% (*n* = 430) at visit 6 to 72% (*n* = 733) at visit 10, our study was able to document the movement through menopausal transition for the majority of the cohort.

Considerable recent research has focused on the association of body mass index (BMI) and percent body fat with VMS and has suggested a positive correlation between increasing BMI and the presence of VMS [30, 32–36]. However, BMI is considered a poor predictor of body mass as it is merely a measure of excess weight and does not distinguish between body fat mass and fat free mass [37]. Our study examined the components of body composition and found lean mass, determined by both fat free mass and skeletal muscle mass, to have a significant effect on the likelihood of developing VMS over time while still considering the potential impact of fat mass. A recent cross-sectional study of 758 women found that trunk lean mass was an independent protective factor for moderate to severe menopausal symptoms and that VMS were independently related to higher BMI and fat mass [38]. The underlying mechanisms of the relationship between body composition and VMS are not entirely clear due to the incomplete understanding of the physiology of VMS [2].

Evidence is emerging on the role of oxidative stress in menopause and its relationship to muscle mass. Lipoperoxide (LPO) levels are considered a measure of oxidative stress and in postmenopausal women are found to be significantly higher than in premenopausal women suggesting that the depletion of estrogen is a risk factor for oxidative stress [39]. A recent study showed that the loss of muscle mass in menopause, due to declining estrogen levels, was negatively associated with oxidative stress (LPO), but skeletal muscle mass was positively associated with serum uric acid which offers a protective role against oxidative stress due to its capacity to clear reactive oxygen species [40]. Increased oxidative stress has been found to also impair the ability of free oxygen radical defenses (FORD) in menopausal women, and that VMS are negatively associated with FORD [41]. Together, these results suggest that declining estrogen in menopause contributes to loss of muscle mass which simultaneously increases oxidative stress and decreases antioxidant levels potentially leading to higher probability of VMS. The results of our study add to the evidence that women with lower levels of LBM are more likely to experience VMS.

Certain limitations need consideration when interpreting results of the current study. The use of BIA measurement involves several assumptions, and while skeletal muscle mass was calculated using a validated equation by Janssen et al. [24] and fat mass and fat free mass were calculated with validated equations by Chumlea et al. [25], this allows for potentially differing interpretation of data supplied by the internal BIA system. Additionally, VMS were assessed through responses to two questions regarding number of days experiencing hot flashes or night sweats in the previous 2 weeks. This data yielded limited information and is subject to recall bias although this population was found to have high specificity and high sensitivity regarding VMS recall [28]. Moreover, odds ratios presented here should not be interpreted as measures of relative risk because VMS are not a rare outcome and the risk may be overestimated.

## Conclusion

This study was among the first to examine the longitudinal association of LBM to VMS, providing new evidence that lean mass may provide protection against the development of VMS as women transition through menopause. Using the longitudinal SWAN database that encompasses a large, multiethnic sample of women from across the United States, these findings are particularly relevant and transferable to general populations in North America. Given the continued resistance to hormone replacement therapy as a means to mitigate symptoms, these results provide for the possibility of symptom prevention through resistance training programs. Importantly, the results of our study suggest that the greatest contributing factor to mitigating symptoms was maintaining LBM throughout the menopausal transition.

## Data Availability

The datasets used during the current study are available on the ICPSR repository https://www.icpsr.umich.edu/icpsrweb/ICPSR/series/00253 [14–19] .

## Abbreviations

BIA: Bioelectrical impedance analysis
BMI: Body mass index
FMI: Fat mass index
FFMI: Fat free mass index
FORD: Free oxygen radical defense
HRQL: Health related quality of life
HT: Hormone therapy
IGF-1: Insulin-like growth factor
IL-6: Interleukine 6
LBM: Lean body mass
LPO: Lipoperoxide
SMI: Skeletal muscle index
SWAN: Study of Women’s Health Across the Nation
TNF-ά: Tumor necrosis factor alpha
VMS: Vasomotor symptoms
